# Reduced shoulder proprioception due to fatigue after repeated handball throws and evaluation of test–retest reliability of a clinical shoulder joint position test

**DOI:** 10.1177/17585732221139795

**Published:** 2022-11-15

**Authors:** Peter Sutton, Marie Lund Ohlsson, Ulrik Röijezon

**Affiliations:** 1 Physiotherapy Department, Karlstad Medical Training Institute, Karlstad, Sweden; 2 Department of Health Sciences, Swedish Winter Sports Research Centre, Mid Sweden University, Östersund, Sweden; 3 Department of Health, Education and Technology, Luleå University of Technology, Luleå, Sweden

**Keywords:** Assessment, fatigue, joint position sense, proprioception, reliability, shoulder

## Abstract

**Background:**

Proprioception is vital for motor control and can be disturbed, for example, due to fatigue or injury. Clinical feasible, reliable and valid tests of shoulder proprioception are warranted. The aim was to investigate the effects of local fatigue on shoulder proprioception and the reliability of a feasible joint position sense test using an experimental repeated measures design.

**Method:**

Forty participants repeated a shoulder joint position sense test to assess test–retest reliability. The test was then utilized on a subgroup of handball players who were subjected to five bouts of a repeated throwing task with the dominant hand. The effect of local fatigue was investigated by comparing the fatigued with the non-fatigued shoulder.

**Results:**

There was a significant interaction for the arm × bout (*p* = 0.028, *η_p_*^2^ = 0.20) and a significant effect for the arm (*p* = 0.034, *η_p_*^2^ = 0.35) with a significant decrease in joint position sense for the throwing arm compared to the non-throwing arm. The intraclass correlation coefficient was 0.78 (95% CI = [0.57; 0.89]). The standard error of measurement between trials was 0.70° (range: 0.57°–0.90°).

**Discussion:**

The results indicate that repeated throwing to fatigue disturbs shoulder joint position sense. Assessment with the modified test showed acceptable reliability and can be a valuable assessment tool in the clinic.

## Introduction

There is a requirement for objective testing and training of proprioception in the clinical setting. However, the assessment of proprioception in the shoulder has traditionally been a challenge.^1^ To date there have been wide variations in methodology and populations sampled with poor clinometric properties reported in tests.^2^ Emerging field-based tests often lack reliability and validity,^3^ while laboratory assessment methods with greater precision and psychometric properties are seldom available for the everyday therapist wanting to assess proprioception.

Proprioception includes kinesthesia (awareness of movement), joint position sense (positional awareness of body parts and angles) and sensation of force, effort and heaviness,^2,4^ and occurs through the stimulation of mechanosensory neurons (termed proprioceptors) located in the tissues providing afferent feedback.^1,4,5^ Through the complex integration of peripheral and central systems,^1,6^ the sum of these proprioceptors and processes are important for both reactive and preparatory control as well as regulating muscle stiffness^4^ in order to achieve coordinated and well-adapted movements and joint stability.^7^ With the shoulder being the most mobile and inherently unstable joint complex in the human body,^8^ the requirement for a strong and coordinated active stabilizing system is imperative. Consequently, any event negatively impacting proprioception has the potential to decrease joint stability, coordination and precision,^7^ whether that be due to disease, pain, effusion, trauma or fatigue.^4^ Studies of the shoulder have shown a reduction in proprioception amongst subjects with joint laxity,^9^ athletes undertaking overhead sports,^10^ in conjunction with shoulder pain,^11^ rotator cuff injuries^12^ and amongst athletes who are fatigued.^7,13–16^ These previous studies have used laboratory equipment and experiments. There is, however, also a need for feasible, swift, valid, and reliable clinical field-based tests.

With the handball shoulder exposed to on average 48,000 throwing motions per season,^17^ 3–12 shots on goal and up to 180 ball contacts per match,^18,19^ local shoulder fatigue may be a concern which in turn may effect shoulder proprioception leading to both reduced performance and increased injury risk. Several mechanisms, both peripheral and central, have been proposed to effect proprioception due to fatigue.^20^ Peripherally, concentric contraction can lead to fatigue through the depletion of metabolic factors with eccentric contractions resulting in both metabolic fatigue and muscle damage, also including delayed onset muscle soreness. This may alter muscle spindle sensitivity, mediated by group III and IV muscle afferents. Central fatigue refers to the reduced maximum voluntary neural drive to the muscle, a central decline in motor unit firing rate.^21^ Central fatigue may lead to an increased sense of effort and altered body schema, or an altered central comparison of the sensory input and the copy of the motor signals (efference copy), leading to disturbed proprioception.^20,22^ Although the exact mechanisms are still unclear, it has been argued that disturbed joint position sense (JPS) due to fatigue is mainly a consequence of central actions and that it is joint-specific.^22^ It has also been proposed that JPS tests using bilateral matching tasks or unilateral pointing tasks involve different central processing.^23^ For the clinical purposes, it is important to assess body parts unilaterally, for example, to compare an injured or fatigued joint to a healthy reference joint, whereby pointing tasks are commonly used.

Contemporary research^3,24^ investigating shoulder JPS has occurred through the use of laser pens enabling assessment with a field-based test.^3,5,24^ Vafadar et al.^24^ investigated the inter-rater, intra-rater reliability and validity of a unilateral active JPS test using the laser pen. They concluded that inter-rater and intra-rater intraclass correlation coefficients were .86 and .78, respectively, for this assessment method and the test had good validity when validated against a Vicon motion-capture system (Vicon, Oxford Metrics Ltd, Oxford, UK). The barrier for clinicians using this method, however, is that although the collection of data is efficient, the calculation of angle error afterwards remains time-consuming. A faster and more feasible way to receive the results would be to use a calibrated target from which the results can be read instantly. The aim of this study was to investigate the effect of fatigue, due to repeated throwing, on shoulder JPS in amateur male handball players. Another aim was to evaluate the test-retest reliability of a modified active JPS test using a laser pen and calibrated two-dimensional (2D) target for swift and feasible calculation of reposition error.

## Materials and methods

### Design

In this study, we used a test–retest and an experimental repeated measures design.

### Participants

Forty participants (15 women and 25 men) were recruited for the assessment of test–retest reliability. This included 27 healthy recreationally active participants and 13 amateur male handball players who subsequently also participated in the second part of the study. Recreationally active were classified as being physically active, as opposed to living a sedentary lifestyle, but not at an elite competing level, as compared to athletes. Amateur handball player was classified as a member of a handball team competing in a sub-elite division whereby handball was not their profession. Participants were aged between 18 and 50 (mean 34.4 years (SD ± 8.4)) and had a mean height of 177.7 cm (± 9.2 cm). Hand dominance was assessed using the Edinburgh handedness inventory^25^ and accordingly one participant was left-handed, 38 were right-handed and one participant was ambidextrous. The amateur male handball players were aged between 18 and 34 (mean 26 (SD ± 4.6 years)) with a mean height of 184.1 cm (± 4.2 cm), mean weight of 91.7 kg (± 10 kg) and had an average playing experience of 14.9 years (± 4.6 years). One player was left-handed, and the remaining participants were right-handed. Participants were included if they reported no history of shoulder injury or if they had returned to competition following an injury and were deemed to be subjectively medically fit. Exclusion criteria for both parts of the trial were current impairment of the shoulder (glenohumeral, scapulothoracic, sternoclavicular or acromioclavicular joints), cervical, thoracic or lumbar spine, if they reported any neurological, systemic or rheumatological dysfunction or if they had any musculoskeletal impairment that would affect their ability to undertake the test. Data collection for 27 participants occurred at a medical training institute with the 13 handball players tested at their handball training facility. Testing of the handball players occurred 48 h after the last match or training session to allow adequate recovery^14^ and was carried out by a Chartered Physiotherapist. Ethical approval was granted from the Swedish ethical review committee (ref nr: 2019-06368) and written consent was obtained from participants to participate in the study.

### Equipment and setup for the active JPS

A tennis elbow support (Mediroyal Nordic AB, Sweden) with a laser pointer (Zhongshan He Tong Optics Electronic Technology LTD, China) attached to the side was strapped to the lateral side of the humerus of participants just proximal to the lateral epicondyle on their dominant arm. Participants sat on a stool without a backrest in front of a laminated movable 2D A3-sized calibrated bullseye target. The participant lifted their dominant arm up to 90° of flexion whilst keeping their elbow extended and wrist in a neutral position with their thumb pointing towards the ceiling. This angle was measured with a goniometer to assure 90° in accordance with Norkin and White.^26^ Whilst maintaining this angle, the target was moved so that the laser dot was pointing at the centre of the target. The participant was then positioned so that the distance from the glenohumeral joint to the centre of the target was 90 cm. The distance of 90 cm was important since the target was calibrated to achieve instant results in degrees using trigonometry, as developed from previous studies using a laser pointer.^24^ For the participants undertaking the throwing task afterwards, this same procedure was then repeated for the non-dominant arm whereby a second target was utilized.

### Active JPS test

**Figure 1.**
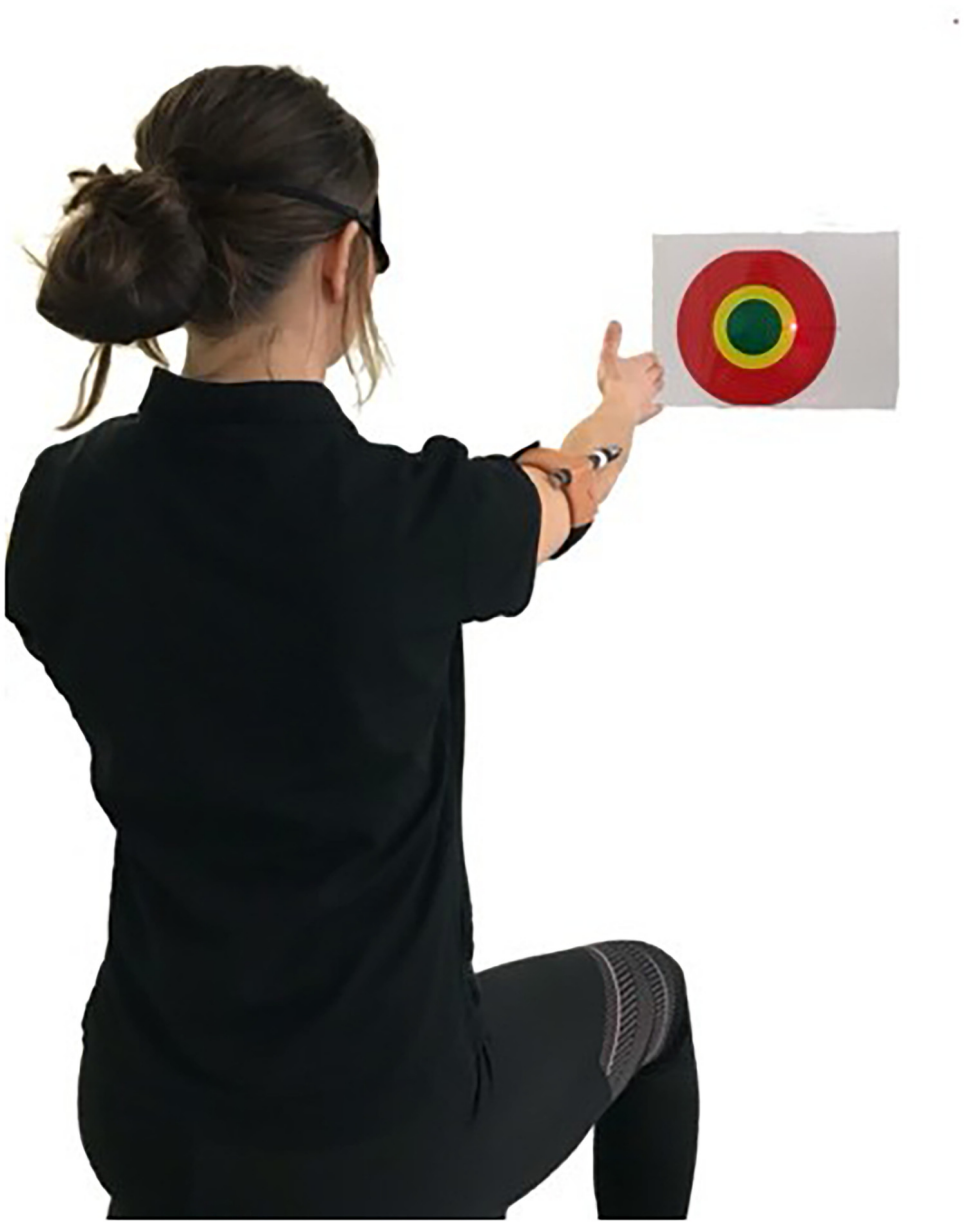
Illustration of the position of the participant, the laser pointer and the target at the reposition angle during the test.

The test was performed as an active-active JPS test with participants blindfolded. On command, the participant actively raised their dominant arm at a comfortable speed to 90° and at this stage, the examiner assisted their shoulder so that the laser was pointing at the centre of the target. When assisting the participant, the examiner held one hand on their forearm and when nearing the target centre, a small oscillation in the sagittal and then the transverse plane was undertaken by the examiner in order to reduce any feedback and thereby minimize learning effects, with the final position resulting in the laser pen pointing at the centre of the target. The participant was instructed to memorize this position for three seconds before lowering their arm to the starting position and then actively raising it at a self-chosen velocity back to the target position. When they felt they had raised their arm to the remembered position, they said ‘yes’ and the angle from the 2D chart was documented (see Figure 1). Participants subsequently repeated four more repetitions, with the examiner assisting the participant's shoulder to 90° each time in conjunction with a small oscillation. Angle documentation occurred through documenting the laser dot on the target (angles on the target ranged from 0° to 9°). In the event of the reproduction angle error exceeding the target maximal error angle of 9°, the angle of error was calculated using trigonometry with tan-1 0 (shoulder angle) = opposite side/adjacent side, where the opposite side was the distance from the recorded laser dot and centre of the target and the adjacent side was the distance from the glenohumeral joint to the centre of the target. Absolute error (AE) was calculated as the mean of the five trials for each test occasion.

### Test–retest reliability

For the assessment of test–retest reliability, participants undertook five repetitions of the active JPS test on their dominant arm. They then rested for 10 min before repeating the test. During this 10-minute period, participants were allowed to leave the test position if they desired.

### Throwing task

Thirteen handballers who had already completed the test–retest reliability trial subsequently participated in the second part of the study. After undertaking their normal warm-up protocol, participants were handed a standard men's handball (size 3, circumference 58–60 cm, weight 425–475 g) from the test leader before advancing to the free throw line (9 m from the goal) and threw the ball towards the goal with their dominant hand. Participants were instructed to throw the ball at maximum speed with a focus on force production as opposed to accuracy. Participants undertook 10 throws with a maximum of ten seconds between each throw and were asked to complete five bouts of the throwing protocol, with a bilateral active JPS test and assessment of fatigue occurring directly after each throwing bout.

### BORG scale

After each throwing bout of 10 throws, participants were asked to rate the level of perceived exertion (RPE) they experienced in their shoulder using the BORG scale (6–20).^27^ Fatigue was defined as reaching 15 or greater and was chosen as it has been shown to correlate with the metabolic responses of fatigue^28^ in accordance with previous research.^16,29^

### Data analysis

The effect of fatigue on JPS AE was analysed with repeated measures ANOVA using IBM SPSS Statistics for Macintosh (Version 25.0. Armonk, NY: IBM Corp). Data for shoulder (dominant and non-dominant), throwing bouts, and shoulder*bout was analysed for all six occasions, that is, baseline and after each of the five throwing bouts, using a mix between (shoulders) and within (bouts) measures design. Post-hoc analysis with Fisher's least significant difference (LSD) was undertaken if the effects were significant for the repeated measures ANOVA.

Test–retest reliability was analysed with an intraclass correlation coefficient (ICC). ICC estimates and their 95% confidence intervals were calculated using IBM SPSS Statistics for Macintosh (Version 25.0. Armonk, NY: IBM Corp) based on a mean rating, absolute agreement and two-way mixed effects model.^30^ For the premise of this study, values were interpreted as follows: less than 0.5 = poor reliability, between 0.5 and 0.75 = moderate reliability, between 0.75 and 0.9 = good reliability, and greater than 0.9 = excellent reliability.^30^

A paired *t*-test was undertaken comparing the test–retest data in order to investigate a possible learning effect. Standard error of measurement (SEM) was calculated using the following formula:

$$\mathrm{SEM}=s_{\mathrm{diff}.}/\surd 2$$

Furthermore, the minimal detectable change (MDC) was calculated for test and retest data using the following formula:

MDC=StandardErrorofMeasurement×1.96×√2

## Results

### Flow of participants through the study

**Figure 2.**
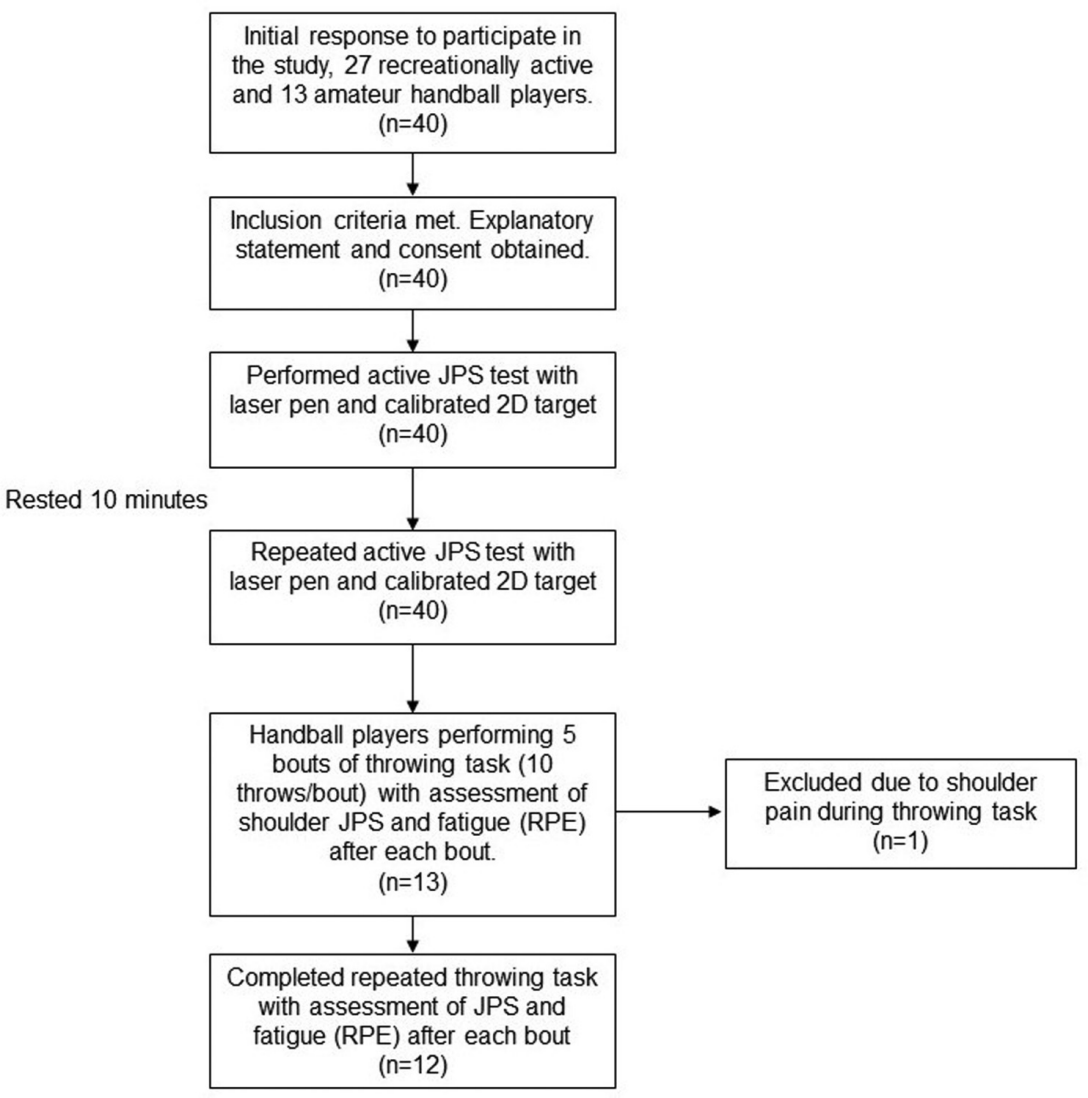
The flow of participants through the study.

JPS: joint position sense; 2D: two dimensional; RPE: rate of perceived exertion.

All forty participants completed the test-retest reliability assessment of the shoulder JPS test with their dominant shoulder. The 13 handball players thereafter continued with the throwing task and subsequent JPS tests of the fatigued and non-fatigued shoulders (Figure 2).

### Effects of fatigue after throwing task

Thirteen players were tested, however, one player failed to complete the throwing protocol due to shoulder pain and subsequently, data analysis occurred for the remaining 12 participants (Figure 2). Assessment of the homogeneity of variance of differences showed the data met the assumption of sphericity using Mauchly's test. The repeated measures ANOVA revealed a significant interaction for arm × bout (*F*5 = 2.74, *p* = 0.028, *η_p_*^2^ = 0.20) and a significant effect for arm (*F*1 = 5.85, *p* = 0.034, *η_p_*^2^ = 0.35) but not for bout (*F*5 = 0.27, *p* = 0.930, *η_p_*^2^ = 0.024). Post-hoc analysis using pairwise comparisons showed a significant difference between the fatigued dominant arm and the non-fatigued non-dominant arm after throwing bout one (*F*1 = 5.72, mean diff 0.64 (95% CI 0.05–1.23), *p* = 0.036, *η_p_*^2^ = 0.34), three (*F*1 = 6.66, mean diff 0.74 (95% CI 0.11–1.37), *p* = 0.026, *η_p_*^2^ = 0.38)) and five (*F*1 = 5.37, mean diff 0.61 (95% CI 0.03–1.19), *p* = 0.041, *η_p_*^2^ = 0.33). There was no significant difference between arms at baseline (*F*1 = 0.10, mean diff 0.06 (95% CI −0.37–0.49), *p* = 0.755, *η_p_*^2^ = 0.01), after bout two (*F*1 = 0.90, mean diff −0.34 (95% CI −1.19–0.50), *p* = 0.392, *η_p_*^2^ = 0.07) and four (*F*1 = 4.18, mean diff 0.86 (95% CI −0.07–1.78), *p* = 0.066, *η_p_*^2^ = 0.28). Table 1 shows data for each arm per test occasion.

**Table 1.** Joint position error for the fatigued dominant and non-fatigued non-dominant shoulder. Measures are absolute error (AE) in mean (sd) for baseline and after throwing bout 1–5, including 95% confidence interval for difference (CI) and *p*-value (Sign.). Rating of perceived exertion (RPE) after each throwing bout is presented as mean (sd).

	Shoulder JPS AE				RPE
	Fatigued	Non-fatigued	CI	Sign.	
*Baseline*	3.2 (0.8)	3.1 (1.1)	−0.37–0.49	*0.755*	*n.a.*
***Bout 1***	**3.7 (0.9)**	**3.0 (0.8)**	**0.05–1.23**	***0.036***	**9.5 (2.5)**
*Bout 2*	3.0 (0.9)	3.3 (0.8)	−1.19–0.50	*0.392*	11.9 (2.2)
***Bout 3***	**3.4 (1.0)**	**2.7 (0.6)**	**0.11–1.37**	***0.026***	**13.7 (2.4)**
*Bout 4*	3.6 (1.3)	2.8 (0.7)	−0.70−1.78	*0.066*	14.9 (2.3)
***Bout 5***	**3.4 (0.9)**	**2.8 (0.8)**	**0.03–1.12**	***0.041***	**15.5 (2.3)**

### Rate of perceived exertion

Subjective assessment of fatigue, assessed with the Borg RPE scale, showed a gradual increase in the level of fatigue after every throwing bout. Table 1 shows the fatigue perceived by participants undertaking the throwing protocol.

### Test–retest reliability

**Figure 3.**
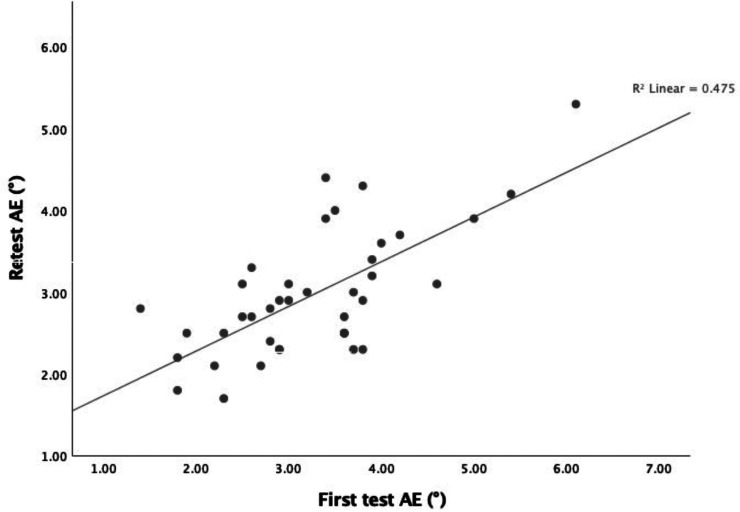
Scatter plot showing absolute error (AE) of the test-retest data with regression line.

The test–retest assessment of the active JPS test revealed an ICC of 0.78 (CI 95% 0.57–0.89). The paired *t*-test showed a significant difference in the JPS test AE scores between the first test (3.27° ± 0.98°) and the retest (2.97° ± 0.78°) (*t*(39) = 2.605, *p* = 0.013). The SEM between the two tests was 0.70° (CI 95% 0.57°–0.90°) with an MDC of 1.9°. A scatter plot of the AE from the test and retest is shown in Figure 3.

## Discussion

The aim of the study was to investigate the effect of local fatigue on shoulder JPS amongst amateur male handball players and evaluate the intra-tester reliability of an active JPS test using a laser pen and calibrated 2D target. The results showed that repeated throwing to fatigue reduced shoulder JPS compared to the control arm and that assessment of JPS using the documented active JPS test had good reliability, with ICC CI 95% ranging from moderate to good reliability.

The data collection for this study was executed in the spring of 2020. Our initial aim was to include all 20 members of the handball team. However, due to the Covid-19 outbreak and related restrictions, we had to stop the data collection earlier. This was very unfortunate, especially regarding the fatiguing part of the study including only 12 participants. This needs to be considered when interpreting the results.

### Throwing task

A reduction in JPS on the fatigued dominant shoulder compared to the contralateral unfatigued control shoulder from baseline to throwing bout five was demonstrated, with post hoc statistical significance after throwing bouts one, three and five. These findings reflect previous research investigating the role of fatigue on shoulder JPS.^7,13–15,29^ However, there was no significant reduction in JPS within the fatigued shoulder over repeated measures in our data, possibly due to the small sample size and a learning effect from repeated measures as discussed under the subheading ‘Test–retest reliability of the active JPS test’. A larger group would have contributed a more reliable result.

Although it has been proposed that contemporary research cannot determine which part of the proprioceptive pathway is responsible for the reduction in proprioceptive sense^31^ the results in the throwing task add weight to the fact that fatiguing effects are joint-specific.^22^ This is shown by the fact that the dominant throwing shoulder showed an increase in AE over the testing bouts compared to the non-dominant non-fatigued control. With the throwing action encorporating both concentric and eccentric components in the shoulder muscles, including the rotator cuff, the depletion of metabolic factors through fatigue can possibly explain the disturbance to JPS witnessed.^20^ The high-frequency throwing protocol may potentially also have created some muscle soreness in the shoulder altering proprioception through the group III and IV muscle afferents.^20^ The impact of throwing influencing JPS via central fatigue is also a possibility. Since this was a unilateral active test, possible mechanisms include failure to adequately drive motor neurons, increased sense of effort and disturbed body schema, and altered central comparison of sensory input and the efference copy of the motor commands.^32^ Investigating peripheral vs central fatigue was, however, beyond the scope of this study but should be explored in future research.

Interestingly, trial 2 and trial 4 did not show significant differences between arms. While the results for trial 4 are in line with the other trials, although not significant (*p* = 0.066), trial 2 confused us. On testing, after throwing bout 2 there was a larger error for the non-throwing arm, although not significantly different (*p* = 0.39). We cannot explain this effect, either physiologically or psychologically. Thixotropy has been highlighted as an important mechanism affecting position sense, especially in passive or weightless tests, and that muscle conditioning should be standardised before testing.^22^ Since we used an active-active JPS test and both the throwing and the test procedures were highly standardised, thixotropy is not a likely explanation for the unexpected results after bout 2. A viable explanation is that it is a random effect. Future research with a larger sample is needed to clarify this.

Some limitations of this study need to be considered in addition to the low number of participants in the fatiguing experiment, as mentioned above. In comparison to handball matches,^18^ participants were exposed to a significantly higher throwing frequency within a short time frame, reinforcing the impact of local fatigue in the shoulder complex which was the target for our study. A further limitation was the absence of measuring the extent of fatigue in the underlying tissues. Since muscle testing with dynamometry (force decline) or EMG (changes in amplitude and frequency due to fatigue) were not included, the true extent of fatigue in the shoulder beyond individual perception cannot be quantified. One can also argue that it would have been more ecologically relevant to assess shoulder JPS in a more throwing-like motion, that is, with an active-active relocation test occurring in abduction and rotation. However, contemporary research^3,5,24^ using a laser pen has only been assessed in flexion. In that respect, we wanted to use a reliable and validated test when assessing shoulder JPS in a field-based situation. Finally, it must be acknowledged that JPS is only one of the elements of proprioception and further work is warranted to investigate the concept of proprioceptive impairment in the throwing shoulder, such as its effects on movement and force sense.

### Test–retest reliability of the active JPS test

The results showed that the assessment of JPS using the documented active JPS test had an ICC of 0.78, which is in line with the findings of Vafadar et al.^24^ Similarly, our research showed a small SEM of 0.7° and an MDC of 1.9°. These results also reflect the findings of Vafadar et al.,^24^ who had an SEM range of 0.6°–1.2° and an MDC of 1.8°. The similarity in results potentially highlights the importance of laser pen placement as methodological variations have occurred between previous studies. Compared to Glendon and Hood^5^ and Balke et al.^3^ who positioned the laser pen on the wrist and index finger respectively, our placement occurred proximally and subsequently interference from the joints distal to the shoulder girdle was eliminated. Although the placement of the laser pen was consistent with that of Vafadar et al.^24^ our methodology differed slightly with the aim to improve the test and clinical feasibility. Participants in our study were seated in order to reduce any sway that has been previously acknowledged.^24^ The current study also utilized a 2D bullseye target. This allowed for AE to be documented and interpreted instantly as opposed to retrospectively using trigonometry. This quick and efficient way of measuring angle error has benefits for clinicians utilizing this assessment method clinically as athletes and patients can get instant feedback on the results. We also incorporated an oscillatory relocation when relocating the shoulder to the target angle to minimize any learning effect as has been reported to influence repeated tests.^33^ However, the learning effect was still evident and is discussed below.

Previously, a variety of methods and equipment have been used to assess shoulder JPS with the greatest intra-rater reliability being demonstrated when using isokinetic dynamometry (ICC 0.92).^1^ Utilizing the documented active JPS test is however similar to that of an inclinometer (ICC 0.84) photo analysis through retrospective angle calculation (ICC 0.81) and is superior to using a goniometer (ICC 0.60).^1^ Although assessment of shoulder and scapula proprioception in the laboratory^34^ has shown a lower SEM, the results in the current study are superior to those reported by Suprak et al.^35^ (SEM range: 1.9°–4.07°) and Kaya et al.^36^ (SEM range: 3.87°–4.5°) who assessed shoulder JPS with digital tracking and isokinetic dynamometry, respectively.^1^

Although acknowledged that the interpretation of the ICC score varies in the literature, the results were interpreted in accordance with the parameters recommended by Koo and Li.^30^ The implication of this is that clinicians are able to assess shoulder JPS with acceptable reliability using the documented methodology. The generalization of results however must be made with caution and further testing for inter-rater and between-days reliability is warranted prior to this assessment method being utilized across multiple clinicians for the same subject. Similarly, the effect of repeated testing needs consideration. When participants were subjected to repeat testing after the throwing task, the reduction in AE in the non-dominant non-fatigued shoulder started to plateau after throwing bout three. An implication of this is that athletes may need to undertake the active JPS test three times before a stable JPS error can be identified. The most likely explanation for this change was a learning effect indicating a systematic bias.^33^ This was also evident in the test-retest trial whereby greater JPS acuity was demonstrated in the retest (−0.3°, SD ± 0.7). This was despite the methodology incorporating an oscillation when relocating the shoulder to minimize feedback and thereby learning effect.

## Conclusion

The findings of the throwing protocol demonstrate a reduction in JPS when the shoulder is fatigued compared to the non-fatigued contralateral shoulder. This has implications for both performance and injury risk during practice and match situations. For example, it may be preferable to avoid a high throwing frequency and essentially high-speed throwing when warming up before matches. Future research should investigate how long the effects of fatigue last and investigate the effects on other elements of proprioception, such as sense of movement and force, effort and heaviness. Test-retest reliability of the shoulder active JPS test has been shown to have good reliability (0.78), with ICC CI95% ranging from moderate to good (0.57–0.89), which is in line with previous research.^24^ This test can therefore be used in the assessment of JPS clinically with acceptable reliability and is quick and feasible to perform. This is important for the identification of disturbed shoulder proprioception in the clinical population to direct rehabilitation interventions.

## Abbreviations

AE: absolute error
ICC: intraclass correlation coefficient
JPS: joint position sense
LSD: least significant difference
MDC: minimal detectable change
RPE: rate of perceived exertion
SEM: standard error of measurement
2D: two dimensional
